# Comparative Analysis of Automated Cloud-Based Mapping and Manual 3D Modeling for AR-Based Indoor Navigation

**DOI:** 10.3390/jimaging12090452

**Published:** 2026-09-18

**Authors:** Evianita Dewi Fajrianti, Amma Liesvarastranta Haz, Yuita Arum Sari, Sritrusta Sukaridhoto, Zacky Maulana Achmad, Rizqi Putri Nourma Budiarti

**Affiliations:** 1 Human Centric Multimedia Research Laboratory, Department of Informatic and Computer Engineering, Politeknik Elektronika Negeri Surabaya, Surabaya 60111, Indonesia; dhoto@pens.ac.id (S.S.); zeckma19@gmail.com (Z.M.A.); 2 Faculty of Computer Science, Brawijaya University, Malang 65145, Indonesia; yuita@ub.ac.id; 3 Department of Information Systems, Faculty of Business Economics and Digital Technology, Universitas Nahdlatul Ulama Surabaya, Surabaya 60237, Indonesia; rizqi.putri.nb@unusa.ac.id

**Keywords:** Augmented Reality, indoor navigation, automated mapping, perceived workload, NASA-TLX

## Abstract

Modern building infrastructures are becoming increasingly complex, creating a need for intuitive indoor navigation systems that can assist users in unfamiliar environments. Augmented Reality (AR) has emerged as a promising solution by providing spatially contextual guidance directly within the user’s field of view. However, many AR indoor navigation systems rely on manually constructed 3D environments, a development process that is time-consuming and prone to spatial inconsistencies with the real-world environment. This study presents a comparative evaluation of two environment creation workflows for AR indoor navigation development: a traditional manual 3D modeling approach and an automated cloud-based spatial mapping workflow using the Immersal SDK. A counterbalanced within-subject experiment was conducted with 48 participants, each of whom completed equivalent indoor navigation development tasks using both workflows in a real-world campus building environment. The development process was divided into three stages: environment acquisition, environment generation, and system integration. Development efficiency was evaluated using stage-based development time measurements, while perceived workload was assessed using the NASA Task Load Index (NASA-TLX). Statistical analysis was performed using repeated-measures analysis to compare workflow performance across development stages. Results show that the automated workflow significantly reduced overall development time by approximately 38% compared to the manual modeling approach, with the most substantial time reductions occurring during the environment acquisition and environment generation stages. NASA-TLX results indicate an approximately 31% reduction in overall perceived workload. Descriptively, the automated workflow had lower mental-demand and effort scores but a higher physical-demand score. A separate researcher-conducted spatial validation of one implementation per workflow showed a higher mean three-dimensional positional error for the automated implementation (22.47 cm) than for the manual implementation (19.14 cm), with a mean paired difference of 3.33 cm across 13 anchor locations. These findings indicate that automated spatial mapping can substantially improve development efficiency and reduce overall perceived workload, while introducing trade-offs in physical demand and spatial alignment accuracy relative to manual environment reconstruction.

## 1. Introduction

As modern indoor infrastructures grow in scale and complexity, traditional navigation tools such as static signage and two-dimensional maps have become increasingly insufficient for navigation guidance [1]. Consequently, the demand for intuitive indoor navigation systems has intensified in order to reduce user confusion and navigation-related frustration [2].

Augmented Reality (AR) has emerged as a promising solution for indoor navigation by overlaying spatially contextual digital guidance directly onto the physical environment [3,4,5]. However, the effectiveness of AR-based navigation systems depends heavily on accurate spatial alignment between virtual content and real-world structures [6]. Achieving such alignment requires reliable spatial representations of indoor environments, typically constructed as three-dimensional models that define the navigable structure of the building [7,8].

Existing AR indoor navigation systems rely on manual 3D modeling workflows to construct these spatial representations [9,10]. In this approach, developers reconstruct building interiors using architectural floor plans or manually measure the physical space and recreate the environment using 3D modeling software before integrating these models into AR development platforms [11]. While this method provides flexibility in defining spatial structures and navigation paths, it introduces significant development effort and depends heavily on the developer’s modeling expertise [8,12]. In addition, discrepancies frequently occur between the reconstructed geometry and the actual physical environment, resulting in scaling mismatches that may negatively affect spatial alignment during AR deployment [13].

Recent advances in visual localization and spatial mapping technologies offer an alternative approach to environment creation for AR applications [14]. In particular, automated cloud-based mapping frameworks such as the Immersal SDK enable the generation of spatial maps directly from captured visual data [15,16]. Instead of manually reconstructing indoor geometry, developers capture images of the environment using a mobile device, which are then processed in the cloud to generate a feature-based spatial map. This approach significantly reduces the need for manual geometry modeling and iterative adjustments, potentially improving development efficiency and simplifying the environment preparation process [7,17,18].

Despite the increasing availability of automated spatial mapping and visual localization technologies, limited empirical research has examined how replacing conventional manual environment modeling with automated image-based mapping affects the AR development process under controlled and functionally equivalent deployment conditions [19]. Existing studies predominantly emphasize localization performance, navigation usability, mapping capability, or individual spatial-computing technologies, while comparatively less attention has been given to the development-stage consequences of selecting different environment-generation paradigms [14]. In particular, the interaction between development efficiency, developer workload, and the spatial alignment quality of the resulting AR environment remains insufficiently characterized across these otherwise separate research strands [7]. This gap is important because the benefits of automation should be evaluated across both human-factor and spatial-performance outcomes rather than inferred from development efficiency alone [20].

To address this gap, this study conducts a controlled comparative evaluation of two distinct environment-generation paradigms within the same AR indoor navigation application: conventional manual 3D modeling and automated cloud-based image mapping. The navigation logic, user interface, target environment, and deployment configuration are maintained across both conditions to support a focused comparison of differences associated with environment preparation while limiting differences in application functionality. The comparison integrates three complementary evaluation dimensions: development efficiency through total and stage-specific completion time, perceived developer workload through the unweighted NASA-TLX (Raw TLX), and spatial alignment quality through repeated three-dimensional positional measurements at predefined physical reference points, hereafter referred to as anchors. This design enables the study to characterize not only the efficiency benefits of automation but also the workload and spatial-alignment trade-offs associated with replacing manual environment modeling with automated visual mapping.

The main contributions of this paper are as follows:

- A controlled, stage-based, within-subject quantitative comparison of two complete environment-preparation workflows of manual 3D modeling and automated image-based spatial mapping, within the same functionally equivalent AR indoor navigation application, while maintaining consistent navigation functionality across both conditions.
- A multidimensional evaluation framework that jointly characterizes workflow efficiency, perceived developer workload, and spatial alignment quality using stage-specific development time, NASA-TLX assessment, and repeated three-dimensional alignment measurements at predefined physical reference points.
- Empirical characterization of the trade-offs introduced by automated spatial mapping, demonstrating that reductions in development time and perceived workload are accompanied by greater physical demand during environment acquisition and a measurable increase in spatial alignment error relative to manual modeling.

## 2. Related Work

This section reviews the existing literature relevant to environment preparation for AR-based indoor navigation, including AR navigation systems, visual localization and image-based spatial mapping, recent developments in three-dimensional scene representation and reconstruction, and human-factor considerations associated with AR development workflows.

### 2.1. AR-Based Indoor Navigation

Augmented Reality (AR) has been widely investigated as an approach to enhance indoor navigation by overlaying digital guidance directly onto the user’s physical surroundings [21]. Numerous systems have demonstrated the feasibility of smartphone-based AR navigation in real environments, integrating spatial tracking with visual directional cues to improve wayfinding efficiency [22,23,24].

Fajrianti et al. [9] implement a mobile AR navigation system that combines visual SLAM and inertial sensing to guide users inside buildings following an initial QR-based localization step. Their work demonstrates practical deployment within campus environments and evaluates system usability through user studies.

Similarly, Huang et al. [25] develop ARBIN, an AR-based navigation system for hospital environments that integrates ARCore with beacon-assisted positioning. The system provides real-time visual guidance and is validated through user testing in a clinical setting.

Extending beyond single-environment deployments, Achmad et al. [15] propose an integrated AR navigation system that supports both indoor and outdoor environments within a unified application. The system enables continuous navigation across multiple buildings and floors without requiring additional hardware.

These studies confirm the practical viability of AR for indoor navigation and demonstrate diverse implementation strategies across application domains. However, all such systems require a prior spatial representation of the indoor environment to enable virtual content placement and navigation guidance. The method used to construct this representation directly influences development effort, scalability, and system configuration.

### 2.2. Environment Representation and Spatial Mapping Approaches

Given the requirement for a preconstructed spatial representation, AR indoor navigation systems adopt different approaches for generating indoor environment models. One approach relies on manually constructed 3D models. Nendya et al. [26] implement AR navigation in Unity by creating a detailed 3D model of the target building and generating navigation paths using NavMesh. This method provides structured path planning but requires manual environment modeling and alignment between virtual and physical spaces.

Structured architectural data has also been used through Building Information Modeling (BIM)-based approaches. Ahn et al. [27] integrate BIM models into AR navigation systems to utilize geometrically accurate and semantically enriched building representations. However, such systems depend on the availability and integration of pre-existing architectural models.

Marker-based and infrastructure-assisted strategies provide an alternative mapping method. Ye et al. [28] anchor spatial content using QR codes as physical reference points for localization. While this reduces the need for full 3D reconstruction, it requires physical installation and configuration within the environment.

More recently, cloud-based spatial mapping frameworks have been adopted to automate environment generation. Achmad et al. [17] integrate the Immersal SDK to perform image-based feature mapping and visual localization, enabling persistent spatial maps without manual geometry reconstruction. This approach shifts environment generation toward computational processing rather than handcrafted modeling.

While the above approaches represent either fully manual or fully automated workflows, intermediate methods have also been explored to balance modeling effort and geometric fidelity. LiDAR-based scanning systems, such as ARKit RoomPlan [29], enable on-device reconstruction of indoor environments using depth sensors, producing structured geometric representations. Similarly, mesh reconstruction and scan-to-BIM techniques generate proxy 3D models from captured spatial data [30]. These hybrid approaches reduce manual modeling effort while preserving explicit geometry.

Spatial mapping and localization frameworks also differ substantially in the type of representation they generate and the role they perform within an AR system. Apple RoomPlan, for example, combines camera observations with LiDAR sensing and machine-learning-based scene understanding to produce structured geometric representations of indoor rooms and multi-room structures. This approach is well suited to applications requiring explicit room geometry, but depends on supported Apple devices equipped with LiDAR sensors [29]. In contrast, ARCore Cloud Anchors use visually derived feature maps to persist and resolve spatial anchors across sessions and devices, primarily supporting persistent or shared AR content rather than reconstructing a complete indoor environment [31]. Azure Spatial Anchors previously provided a similar cross-platform persistent-anchor service, although the service was retired by Microsoft on 20 November 2024 [32]. These approaches therefore address related but distinct spatial-computing requirements and are not direct functional equivalents of feature-map-based indoor visual positioning systems.

The Immersal-based workflow examined in this study occupies a different position within this design space. Rather than generating an explicit parametric room model or maintaining only individual persistent anchors, it constructs a feature-oriented visual map from captured images that can subsequently be used for camera relocalization and spatial registration. This characteristic allows the mapping process to operate using conventional camera-equipped mobile devices without requiring dedicated depth sensing hardware, while shifting environment-map generation toward cloud-based image processing. Table 1 summarizes these differences in terms of input modality, spatial representation, primary role, and implementation considerations.

Although existing studies primarily evaluate localization accuracy, usability, or system integration, limited work examines how different spatial mapping strategies influence development efficiency and implementation complexity in AR indoor navigation systems [14,19].

### 2.3. Visual Localization and Modern Scene Representation

Beyond application-specific AR navigation systems, visual localization and image-based mapping constitute fundamental components of contemporary spatial computing pipelines. Visual localization estimates the position and orientation of a camera relative to a previously established representation of the environment. Recent reviews distinguish approaches based on image and feature correspondences, structure-based localization, and learned camera-pose estimation, while visual SLAM systems jointly address camera motion estimation and environmental mapping through interconnected front-end, optimization, loop-closure, and mapping components [14,34,35]. These developments are particularly relevant to image-based AR mapping systems because captured visual observations support both environmental representation and subsequent camera relocalization.

Recent imaging research has also expanded the forms in which three-dimensional scenes can be represented. Neural Radiance Fields (NeRFs) encode scene appearance and volumetric density as a continuous neural function learned from multi-view images with known camera poses [36]. More recently, 3D Gaussian Splatting (3DGS) has introduced an explicit radiance-field representation based on spatial Gaussian primitives, enabling high-quality novel-view synthesis and supporting applications such as scene reconstruction and editing [37,38]. Modern image-based reconstruction further encompasses depth maps, voxels, point clouds, meshes, and learned implicit surfaces [39], while scan-to-BIM pipelines automate the conversion of captured spatial observations into structured geometric models [18,30].

These approaches differ in their representation objectives and outputs. Dense reconstruction and neural scene representations generally emphasize geometric reconstruction or photorealistic view synthesis, whereas feature-oriented visual maps primarily support camera relocalization and spatial registration. The Immersal-based workflow evaluated in this study belongs to the latter category and should therefore not be interpreted as producing a dense mesh, NeRF, Gaussian-splat representation, or explicit polygonal building model.

### 2.4. Developer Workload in AR and Spatial Systems

Workload evaluation is widely used in immersive-systems research to characterize the perceived demands associated with interactive tasks. Criollo-C et al. [40] applied the NASA Task Load Index (NASA-TLX) to measure perceived mental workload in virtual reality educational environments, illustrating its application to the evaluation of workload in immersive interaction.

Workload differences have also been examined in AR authoring contexts. Brata et al. [41] compared in-situ mobile and desktop-based tools for location-based AR content creation, measuring task completion time and NASA-TLX scores. Their results showed significant differences in perceived mental effort between authoring workflows.

From a human factors perspective, Ashtari and Chilana [42] reported that new AR developers encounter challenges related to spatial reasoning and three-dimensional interaction, indicating that AR development introduces cognitive complexity beyond conventional 2D software tasks.

Similarly, Baig and Kavakli [43] analyzed cognitive load during 3D modeling using keyboard/mouse and multimodal inputs, employing EEG signals and questionnaires. Their findings revealed variations in neural activity across interaction modalities, indicating that interaction modality can influence cognitive demand during spatial modeling.

Despite these investigations, existing studies have primarily examined end-user interaction, AR authoring, or general spatial-modeling tasks. Comparatively limited attention has been given to perceived developer workload when alternative environment-generation workflows are used to implement the same AR indoor navigation functionality.

## 3. Environment Creation Workflows for Comparison

This study evaluates two environment-creation workflows within the same AR indoor navigation system. The system was based on a previously developed framework [44]. To support functional comparability, both workflow conditions used the same Unity-based AR navigation application, navigation logic, user interface, interaction components, navigation destinations, target indoor environment, and deployment configuration. The primary distinction between the conditions was the method used to create and spatially align the environment representation. The manual workflow used physical measurements and explicit polygonal 3D modeling, whereas the automated workflow used cloud-generated feature-based mapping and visual relocalization. The resulting representation from each workflow was integrated into the same navigation application to support the predefined AR navigation functions. Accordingly, the comparison examined development time, perceived developer workload, and spatial alignment quality while maintaining the same application-level functionality across both conditions.

### 3.1. Workflow Selection Rationale

This study compares manual 3D modeling and cloud-based image mapping as two alternative environment-preparation workflows within a practical AR development context. The workflow selection supports a focused evaluation of development efficiency, perceived developer workload, and spatial alignment quality rather than optimization for maximum geometric reconstruction fidelity.

The automated workflow employs the Immersal SDK, which generates a feature-oriented spatial map from captured camera images through cloud-based processing. In the present setup, this camera-based approach supported visual relocalization without requiring a specialized depth sensor. The manual workflow employs conventional 3D modeling based on physical measurements and floor-plan references, representing an established environment-preparation approach in AR development.

Although the workflows produce different spatial representations, both were used to implement the same predefined AR navigation functions under the same application and deployment configuration. This design improves comparability between the workflow conditions without assuming equivalence between the resulting spatial representations.

Alternative spatial-computing approaches, including LiDAR-assisted room reconstruction, local SLAM, persistent spatial-anchor services, and on-device depth sensing, involve different trade-offs in hardware requirements, spatial representation, processing location, and intended use. These alternatives are discussed in the Related Work section to position the Immersal-based workflow within the broader mapping and localization landscape. The present experiment does not benchmark these technologies directly; instead, it provides a focused comparison between conventional manual environment modeling and automated image-based visual mapping within the same AR indoor navigation application.

### 3.2. Manual 3D Modeling Setup

The manual workflow comprised three stages: environment acquisition, environment generation, and application integration. During environment acquisition, participants collected floor-plan references and recorded the required physical dimensions using a laser range finder and tape measure. During environment generation, these references were used to manually construct an explicit three-dimensional representation of the indoor environment in Blender. Geometric elements, including walls, corridors, doors, and staircases, were modeled to approximate the physical building layout.

**Figure 1:**
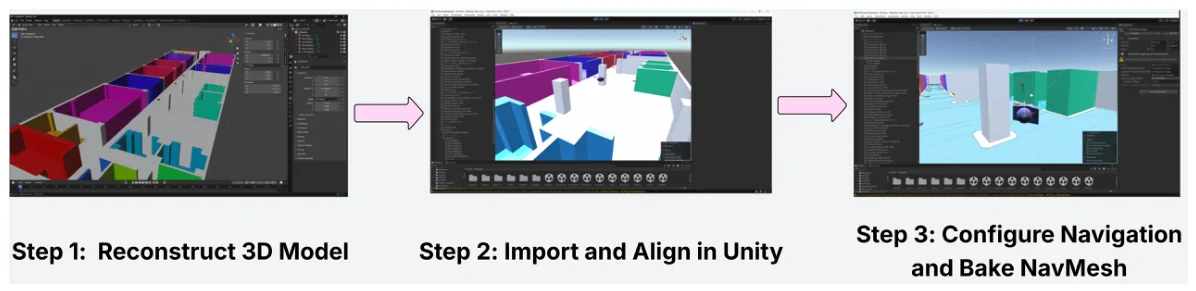
Manual 3D modeling workflow, comprising on-site environment measurement, construction of an explicit 3D model, spatial alignment in Unity, and configuration of the predefined navigation paths.

During application integration, the completed 3D model was exported from Blender and imported into Unity. Participants adjusted its scale and coordinate alignment to match the virtual representation with the physical environment. Navigation paths were then configured using Unity’s NavMesh system, while navigation destinations and starting points were positioned relative to the modeled geometry. The predefined navigation logic used these points to provide spatially aligned guidance within the target indoor environment. The overall manual workflow is illustrated in Figure 1.

**Figure 2:**
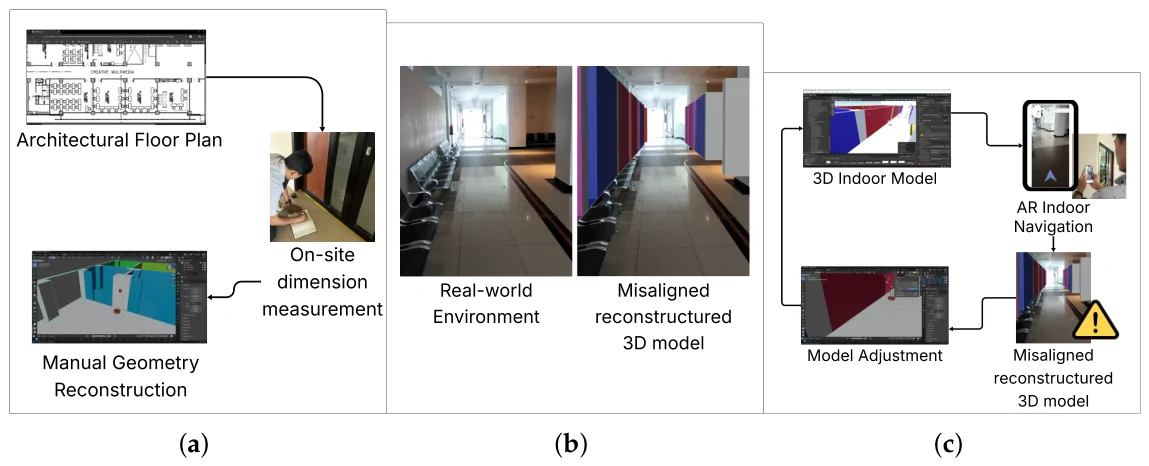
Illustration of challenges associated with the manual environment-modeling workflow: (**a**) the indoor environment is modeled using floor-plan references and physical measurements; (**b**) discrepancies between the modeled geometry and physical dimensions can produce spatial misalignment during AR deployment; and (**c**) identified alignment discrepancies may require model adjustment and repeated deployment.

The manual workflow could require iterative adjustments during application integration when discrepancies were identified between the modeled geometry and the physical environment. These adjustments included refining the model geometry or scale, realigning the model in Unity, and redeploying the application to verify the resulting spatial alignment. Figure 2 illustrates the principal challenges associated with this process.

### 3.3. Automated Cloud-Based Mapping Setup

The automated workflow comprised environment acquisition, cloud-based map generation, and application integration. During environment acquisition, participants used the default RGB camera of a Samsung Galaxy A56 smartphone to capture 70–100 images of the target indoor environment. Participants walked through the environment and captured the scene from multiple viewpoints to obtain sufficient visual coverage for subsequent map generation. No specialized depth sensor was used in this workflow.

During map generation, the captured image set was uploaded to the Immersal cloud service through a Wi-Fi connection with measured network speeds of approximately 6–17 Mbps. The service processed the uploaded visual observations and generated a feature-oriented spatial map for visual relocalization. The generated map was considered ready for integration when it became available in the Immersal dashboard and could be imported into Unity.

During application integration, the generated spatial map was imported into the Unity-based AR navigation application. The system performed visual relocalization by matching live camera observations against the stored feature map and estimating the position and orientation of the mobile device relative to the mapped environment. This process provided the spatial reference required to align the virtual navigation content without using an explicitly modeled polygonal representation of the building.

**Figure 3:**
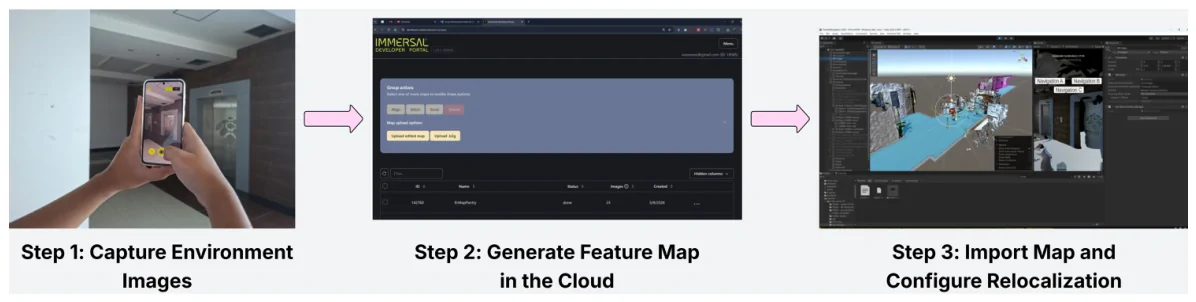
Automated cloud-based mapping workflow, comprising image acquisition using a mobile device (**left**), cloud-based spatial-map generation and availability through the Immersal dashboard (**center**), and integration of the generated feature-oriented map into the Unity-based AR navigation application for visual relocalization and deployment (**right**).

The automated condition used the same navigation destinations, path-guidance functions, user interface, and interaction logic as the manual condition. The workflow-specific spatial map, relocalization configuration, and spatial alignment procedures differed from those used in the manual workflow. The automated mapping workflow was implemented using Unity 2022.3.30f1, AR Foundation 5.16, and Immersal SDK 2.11. The principal stages of the workflow are illustrated in Figure 3.

## 4. Experimental Methodology

This section describes the experimental design and measurement procedures used to compare the manual 3D modeling and automated cloud-based mapping workflows. The evaluation comprised three dimensions: development efficiency based on total and stage-specific completion time, perceived developer workload assessed using NASA-TLX, and spatial alignment quality evaluated through repeated three-dimensional positional measurements. The experiment maintained the same navigation functions, user interface, target environment, and deployment configuration across both workflow conditions while varying the environment-preparation method.

### 4.1. Participants

The study involved 48 participants recruited from a multimedia broadcasting course with prior experience in AR and 3D development. Participants ranged in age from 18 to 20 years (Mean = 19.0, Standard Deviation (SD) = 0.89). The sample consisted of 33 males (68.8%) and 15 females (31.2%).

All participants had programming experience ranging from 1 to 2 years. Regarding AR development experience, 37 participants (77.1%) reported 1 year of experience with Unity-based AR development, while 11 participants (22.9%) reported 2 years of experience. Most participants had prior exposure to Unity-based AR development and 3D modeling workflows, while familiarity with the Immersal SDK varied across participants.

Before the experiment, all participants received two separate standardized 30 min training sessions, with one session provided for each workflow. The manual-workflow training covered on-site measurement, 3D environment modeling in Blender v5.0, Unity integration, spatial alignment, and NavMesh configuration. The automated-workflow training covered image-acquisition procedures, Immersal cloud-based map generation, visual-relocalization configuration, and spatial-map integration into Unity. No separate practice task was administered. Both training sessions were completed before the timed experimental tasks, and the training durations were not included in the recorded development time.

The training was intended to reduce procedural unfamiliarity before the timed task. Nevertheless, differences in participants’ prior experience with Unity, 3D modeling, and image-based mapping could not be eliminated completely and were considered when interpreting the results. All participants provided informed consent before participating in the study.

### 4.2. Experimental Design

In each condition, participants were required to prepare a digital representation of the same target indoor environment and integrate it into the same predefined AR indoor navigation system using the assigned workflow. The functional requirements of the resulting navigation application were kept constant across conditions; only the environment creation and spatial alignment procedures differed between the manual and automated workflows.

To minimize learning effects and order bias, each participant was assigned a unique identification number. The participant identifiers were randomized using an online random-number generator and divided into two equally sized workflow-order groups (n=24 per group). Group A completed the manual workflow followed by the automated workflow (A→B), whereas Group B completed the workflows in the reverse order (B→A). This counterbalancing strategy was intended to reduce potential learning, fatigue, and sequence effects associated with repeated exposure to the development tasks.

Each participant completed both workflow conditions on the same day. Each condition was initiated as a new workflow, and participants were not permitted to reuse measurements, 3D models, image sets, scans, maps, or other outputs from their preceding condition or from another participant. Although this procedure maintained independence between the workflow outputs, familiarity with the target environment or experimental procedure acquired during the first condition could still influence performance in the second condition. Balanced workflow ordering and random assignment were used to reduce such order effects; however, a dedicated statistical analysis of workflow order was not performed. Consequently, residual effects associated with prior experience, learning, fatigue, or workflow sequence cannot be excluded. Investigating workflow order effects more formally represents an important direction for future work.

The hardware, software, experimental protocol, and target indoor environment were standardized across the experimental sessions. The automated mapping workflow was performed using identical mobile devices (Samsung Galaxy A56, Samsung Electronics, Suwon, South Korea), while spatial measurements in the manual workflow were obtained using identical laser range finders (KEELAT KLDM02, KEELAT SDN. BHD. Petaling Jaya, Malaysia) and tape measures. Due to the large number of participants, data collection was distributed across multiple days, with a maximum of eight participants completing the experiment per day. Within each daily session, two participants performed the development task concurrently under the supervision of experiment observers.

**Figure 4:**
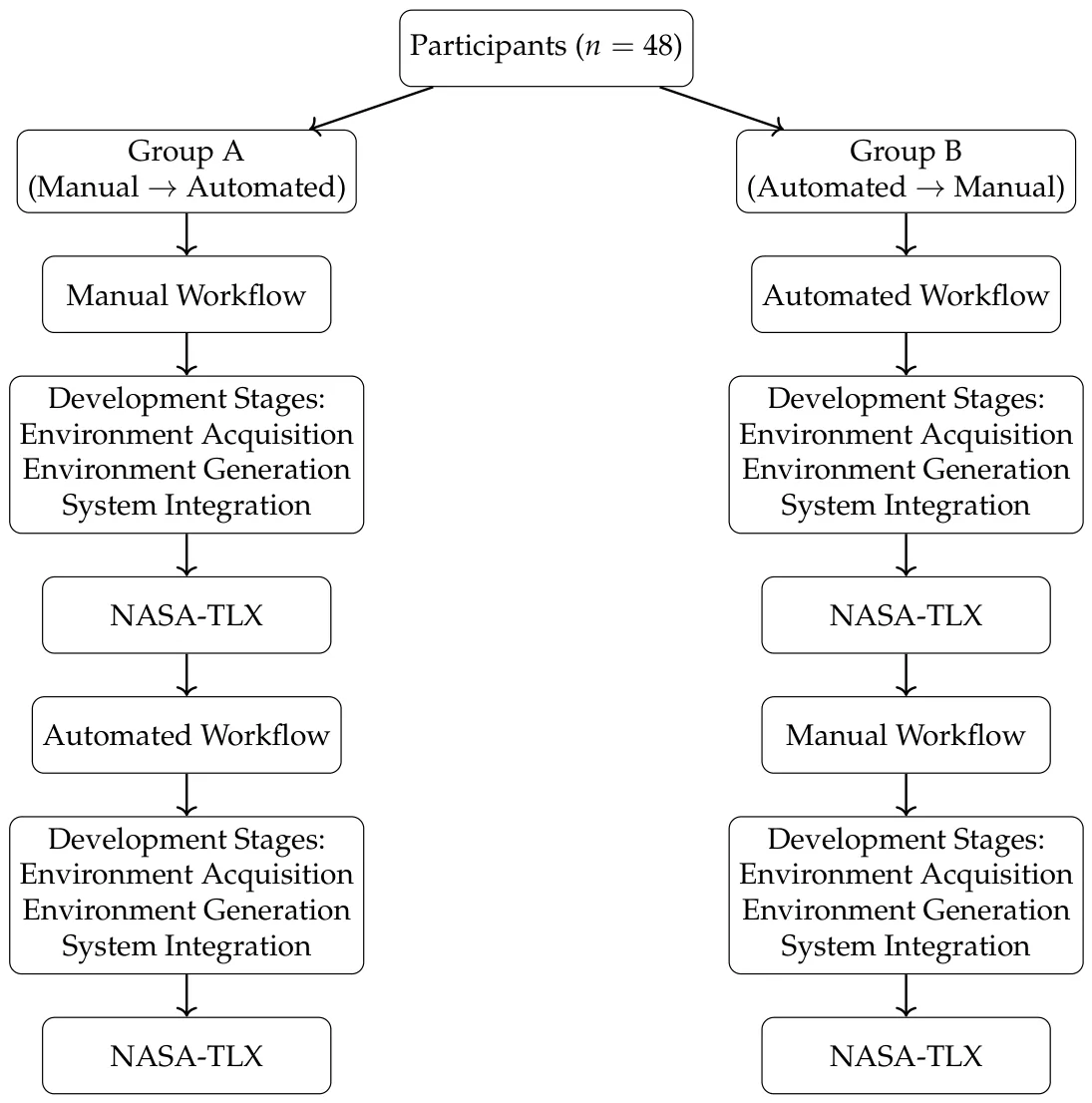
Counterbalanced within-subject experimental design. All participants completed both workflows using one of two randomized workflow orders. Each workflow was initiated from the beginning without reusing measurements, models, images, maps, or other outputs from the preceding condition. Development time was recorded across the three workflow stages, and NASA-TLX was administered after each condition.

The independent variable in this study was the environment generation workflow. The participant-level outcomes comprised total and stage-specific development time and perceived workload measured using the NASA Task Load Index (NASA-TLX). A separate researcher-conducted validation evaluated the spatial alignment quality of one implementation generated specifically for each workflow. All other system components were held constant to improve comparability between the two workflow conditions and support a focused comparison of the outcomes associated with each workflow. Figure 4 illustrates the experimental procedure and counterbalanced workflow order used in the study.

### 4.3. Development Task

**Figure 5:**
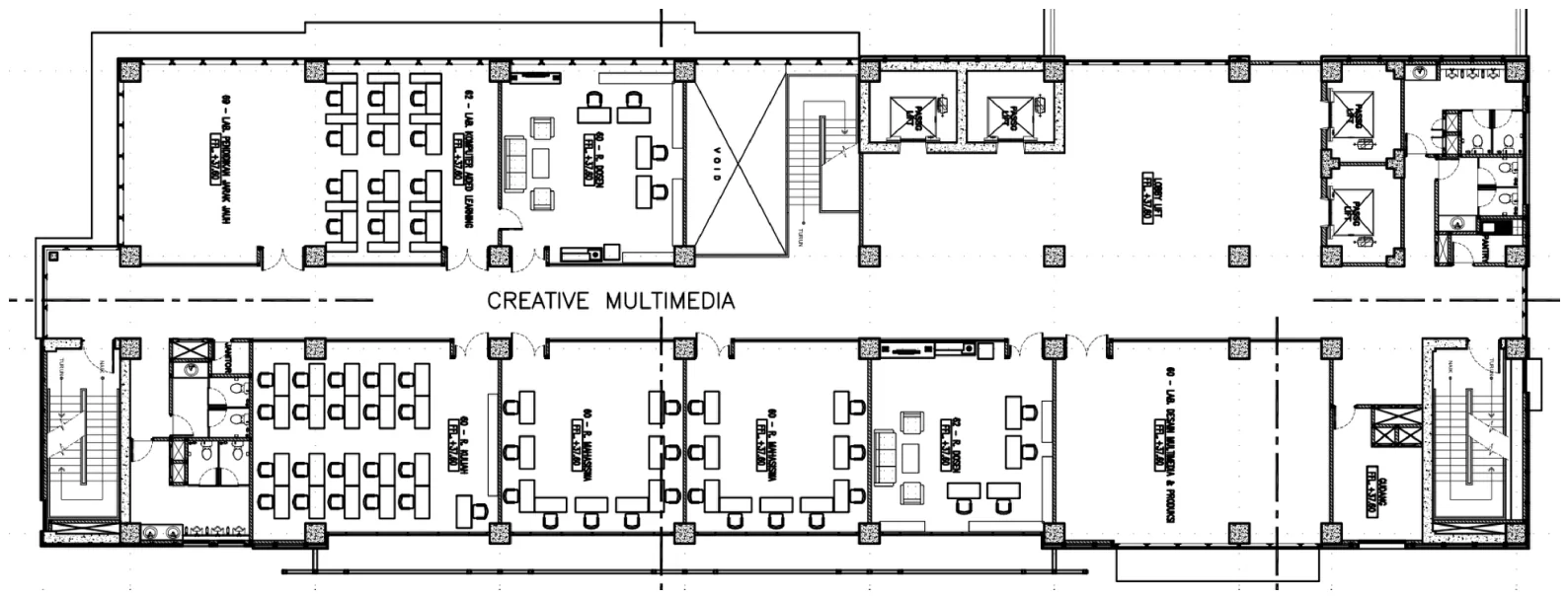
Floor layout of the ninth floor used as the experimental environment, covering approximately 1084 $m^{2}$.

In each experimental condition, participants prepared a digital representation of the target indoor environment using the workflow specified for that condition and integrated the resulting representation into the predefined AR indoor navigation application. The target environment was the ninth floor of the Graduate Building at Politeknik Elektronika Negeri Surabaya (PENS), covering an area of approximately 1084 $m^{2}$. The floor contained corridors, rooms, and structural elements representative of the tested indoor navigation scenario. Its layout is shown in Figure 5.

To support stage-specific evaluation of development efficiency, the environment-preparation process was divided into environment acquisition, environment generation, and system integration. Development time was recorded separately for each stage by trained experiment observers using stopwatches and standardized timing sheets. Only intervals during which participants were actively performing workflow-related tasks were accumulated. Passive delays attributable solely to network transmission or server-side map processing were excluded; consequently, the recorded durations represent active workflow time rather than complete end-to-end elapsed time.

For the *environment-acquisition* stage, timing began when participants started collecting the required environmental information and ended when the required measurements or image set had been completed. In the manual condition, participants used a KEELAT KLDM02 laser range finder, a tape measure, and floor-plan references to record the required physical dimensions. In the automated condition, participants used a Samsung Galaxy A56 smartphone to capture the prescribed image set from multiple viewpoints. Physical measurement and recording activities, additional image capture, rescanning, and active work associated with unsuccessful or repeated acquisition attempts were included in the recorded time.

For the *environment-generation* stage, manual-workflow timing began when participants started constructing the indoor environment model in Blender and ended when the completed 3D model was ready for import into Unity. Automated-workflow timing began when participants initiated submission of the captured image set to the Immersal cloud service. The stage was considered complete when the generated feature-oriented spatial map became available in the Immersal dashboard and was ready for Unity import. Active preparation, submission, and work associated with unsuccessful or repeated attempts were included, whereas passive network-transmission and server-processing intervals were excluded.

For the *system-integration* stage, timing began when participants started importing the generated environment representation into the Unity project and ended when the complete AR navigation application could be successfully run. In the manual condition, participants imported the polygonal 3D model, performed spatial alignment, and configured the predefined navigation paths using Unity’s NavMesh system. In the automated condition, participants imported the generated spatial map and configured visual relocalization for AR deployment. The recorded integration time included spatial configuration, navigation setup, debugging, correction of representation-related problems, application building, and active work associated with repeated deployment attempts required to obtain a functioning navigation application.

Two participants could perform their development tasks concurrently during an experimental session, with trained observers recording each participant’s timing separately. The total development time for each workflow condition was calculated as the sum of its three active stage durations. After completing each workflow condition, participants completed the NASA-TLX questionnaire to evaluate the perceived workload associated with that workflow.

### 4.4. Evaluation Metrics and Statistical Analysis

Development efficiency was evaluated using stage-specific and total development time. Development time was recorded separately for environment acquisition, environment generation, and system integration. The total development time for each participant and workflow condition was calculated as the sum of the three active stage durations.

Perceived workload was evaluated using the unweighted Raw NASA Task Load Index (Raw TLX). After completing each workflow condition, participants rated six workload dimensions: mental demand, physical demand, temporal demand, performance, effort, and frustration. No pairwise subscale-weighting procedure was administered. For each participant and workflow condition, the overall Raw TLX score was calculated as the arithmetic mean of the six subscale ratings. Higher overall scores represented greater perceived workload. For the performance dimension, higher scores represented poorer perceived performance. The six subscale scores were summarized descriptively, whereas inferential workload analysis was performed using the participant-level overall Raw TLX score.

Because all participants completed both workflow conditions, participant-level comparisons between the manual and automated workflows were treated as paired. Total development time was compared between workflows using a paired-sample *t*-test. The mean paired difference was reported with its 95% confidence interval, and the standardized paired effect size was calculated using Cohen’s $d_{z}$.

Stage-specific development time was analyzed using a two-way repeated-measures ANOVA with workflow and development stage as within-subject factors. Sphericity was assessed using Mauchly’s test, with Greenhouse–Geisser correction applied when required, and effect sizes were reported using partial eta squared ($\eta_{p}^{2}$). A significant interaction was followed by stage-specific paired-sample *t*-tests with Bonferroni correction and Cohen’s $d_{z}$.

Participant-level overall Raw TLX scores were compared between the manual and automated workflows using a paired-sample *t*-test. The mean paired difference was reported with its 95% confidence interval, and the standardized paired effect size was calculated using Cohen’s $d_{z}$. For the paired-sample analyses, normality was assessed for the corresponding participant-level paired difference scores using the Shapiro–Wilk test. Statistical significance was evaluated using a two-sided significance level of α=0.05.

As a post-hoc exploratory analysis, participant-level workflow-benefit scores were calculated for total development time and overall Raw TLX by subtracting the automated-workflow value from the corresponding manual-workflow value. Positive benefit scores represented development-time savings or reductions in overall perceived workload associated with the automated workflow. The benefit scores were compared according to AR/Unity experience (two years versus one year) and prior 3D modeling experience (yes versus no). Because the resulting experience subgroups were independent and unequal in size, two-tailed Welch independent-samples *t*-tests were used. The Holm procedure was applied across the four exploratory comparisons to control the family-wise error rate. These analyses were interpreted as exploratory because participant experience was not the primary experimental factor and the sample represented a restricted range of experience.

### 4.5. Spatial Alignment Validation

To complement the participant-based evaluation of development time and perceived workload, a separate spatial alignment validation was conducted by the research team. This validation was not performed by the 48 study participants and was analyzed independently from the participant-level development-time and Raw NASA-TLX measurements. For this validation, the research team created one manual 3D model implementation and one automated Immersal-map implementation specifically for spatial testing, following the workflow configurations described previously.

Thirteen predefined anchor points were distributed across navigation-relevant locations in the target indoor environment, including door centers, corridor intersections, wall corners, and navigation turning points. These locations were selected to sample different structural features and spatial regions encountered during indoor navigation rather than to represent every possible location within the environment.

**Figure 6:**
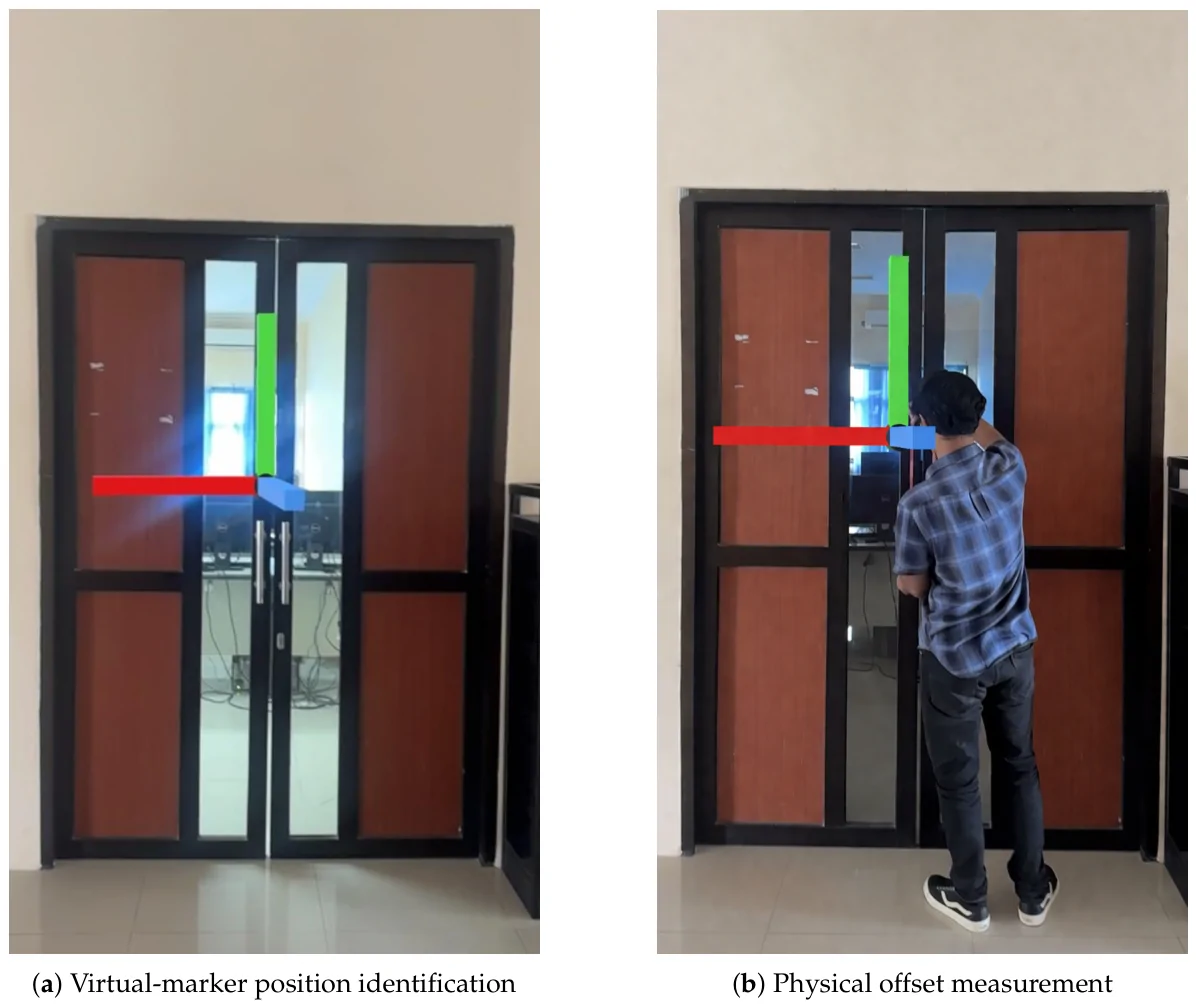
Spatial alignment validation procedure: (**a**) identification of the perceived virtual-marker position relative to a predefined physical reference point and (**b**) measurement of the physical offsets between the perceived virtual-marker position and the corresponding reference point. Note: The red, blue, and green arrows represent the *x*, *y*, and *z* axes, respectively.

A two-observer measurement protocol was used. At each anchor, the first observer viewed the AR scene through the mobile device and identified the perceived position of the virtual marker relative to the corresponding predefined physical reference point. The second observer measured the offsets between the perceived virtual-marker position and the physical reference point along three orthogonal spatial axes using a tape measure. The same anchor locations and measurement procedure were used for the manual and automated implementations. The measurement procedure is illustrated in Figure 6.

Each anchor was evaluated through five repeated localization or alignment attempts under each workflow condition. Before each repetition, a new localization or spatial-alignment attempt was performed. After the spatial reference had been re-established, the positional offsets were measured again using the same two-observer procedure. This process produced 65 measurements per workflow implementation and 130 measurements in total.

For each repetition, the three-dimensional positional error was calculated from the measured axis offsets as

(1) $$E_{3D}=\sqrt{{\left(\Delta x\right)}^{2}+{\left(\Delta y\right)}^{2}+{\left(\Delta z\right)}^{2}},$$

where Δx, Δy, and Δz denote the measured positional offsets, in centimeters, along the three spatial axes. Accordingly, $E_{3D}$ is expressed in centimeters.

For inferential analysis, the five repeated measurements were averaged within each anchor and workflow implementation. This aggregation produced 13 paired anchor-level mean errors, with the predefined anchor locations serving as the units of inferential comparison. Normality of the 13 paired difference scores was assessed using the Shapiro–Wilk test. The anchor-level mean errors of the two implementations were compared using a paired-sample *t*-test. The mean paired difference was reported with its 95% confidence interval, and statistical significance was evaluated using a two-sided significance level of α=0.05.

## 5. Results

This section presents the findings from two complementary evaluation components. First, the counterbalanced within-subject participant experiment (n=48) compared the manual and automated workflows in terms of total and stage-specific development time and perceived workload measured using the Raw NASA Task Load Index (Raw TLX). Second, the separate researcher-conducted spatial validation compared the spatial alignment errors of the two implementations generated specifically for this validation at 13 predefined anchor locations.

The results are presented in five parts: total development time, stage-specific development time, perceived developer workload, spatial alignment validation, and a post-hoc exploratory analysis of workflow benefits according to participant experience.

### 5.1. Development Time Comparison

Total development time was calculated for each participant and workflow condition as the sum of the *environment-acquisition*, *environment-generation*, and *system-integration* durations. Table 2 summarizes the participant-level total development times for the manual and automated workflows.

Normality of the participant-level paired difference scores was assessed using the Shapiro–Wilk test. The test did not indicate a departure from normality (W=0.989, p=0.930). A paired-sample *t*-test was therefore used to compare total development time between the two workflows.

The manual-minus-automated paired comparison showed that the automated workflow required significantly less total development time than the manual workflow, t(47)=24.59, p<0.001. The mean paired reduction was 101.17 min (95% CI [92.89, 109.44]), corresponding to approximately 38.3% of the mean manual-workflow duration. The standardized paired effect size was large ($d_{z}=3.55$).

**Figure 7:**
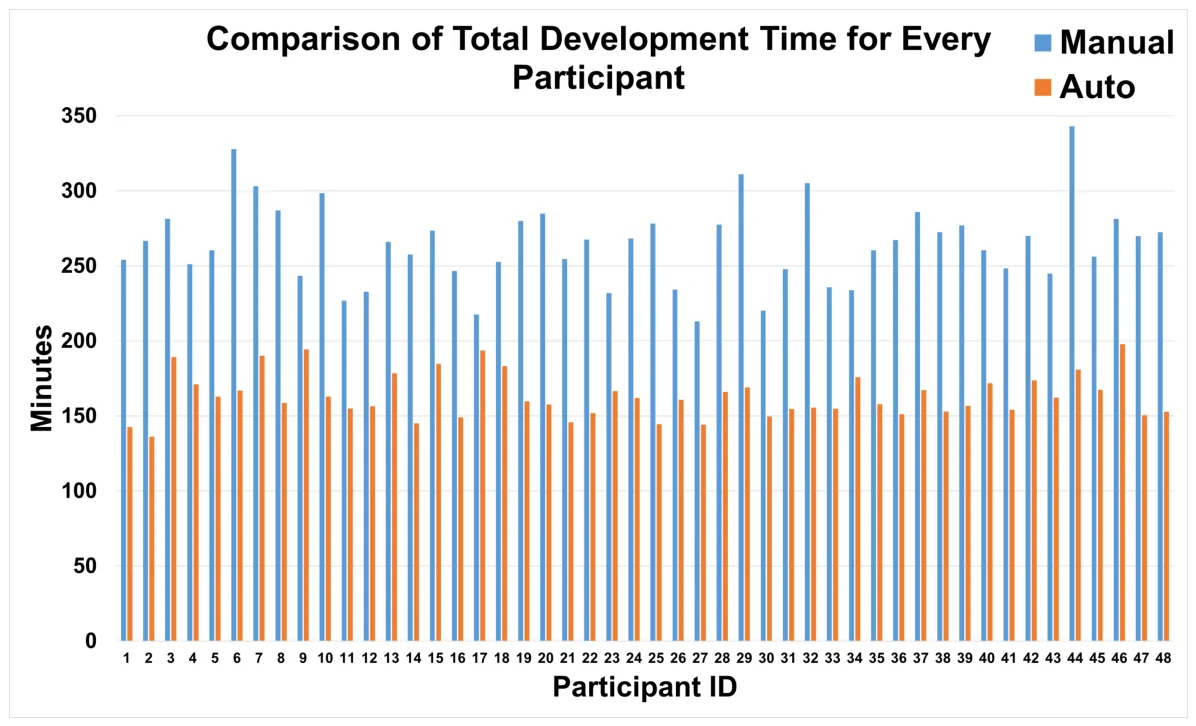
Participant-level comparison of total development time between the manual 3D modeling and automated cloud-based mapping workflows.

Figure 7 presents the participant-level total development times under both workflow conditions. The participant-level pattern was consistent with the aggregate paired comparison, with the automated workflow requiring less development time for nearly all participants.

### 5.2. Stage-Based Development Time Analysis

Development time was analyzed across three stages: environment acquisition, environment generation, and system integration. Table 3 presents the participant-level mean and standard deviation for each workflow and development stage.

Descriptively, the largest reduction occurred during environment acquisition, followed by environment generation. The difference between workflows was smaller during system integration.

A two-way repeated-measures ANOVA was conducted with workflow type (manual and automated) and development stage (environment acquisition, environment generation, and system integration) as within-subject factors. Mauchly’s test indicated that the sphericity assumption was violated for the development-stage effect (W=0.828, $\chi^{2}\left(2\right)=8.50$, p=0.014) and the workflow-by-stage interaction (W=0.828, $\chi^{2}\left(2\right)=8.49$, p=0.014). Greenhouse–Geisser corrections were therefore applied to both effects (ϵ=0.853 and ϵ=0.854, respectively).

The analysis showed a significant main effect of workflow, F(1,47)=604.74, p<0.001, $\eta_{p}^{2}=0.928$. A significant main effect of development stage was also observed, F(1.71,80.22)=166.18, p<0.001, $\eta_{p}^{2}=0.780$. The workflow-by-stage interaction was significant, F(1.71,80.23)=124.82, p<0.001, $\eta_{p}^{2}=0.726$, indicating that the magnitude of the workflow difference varied across the three development stages.

Bonferroni-adjusted paired comparisons showed significantly lower development times under the automated workflow at all three stages. The mean manual-minus-automated difference was 58.71 min for environment acquisition, t(47)=20.22, adjusted p<0.001, $d_{z}=2.92$; 33.13 min for environment generation, t(47)=17.81, adjusted p<0.001, $d_{z}=2.57$; and 9.33 min for system integration, t(47)=4.97, adjusted p<0.001, $d_{z}=0.72$. Thus, although the automated workflow produced a statistically significant reduction at every stage, its efficiency advantage was substantially smaller during system integration than during environment acquisition and generation.

**Figure 8:**
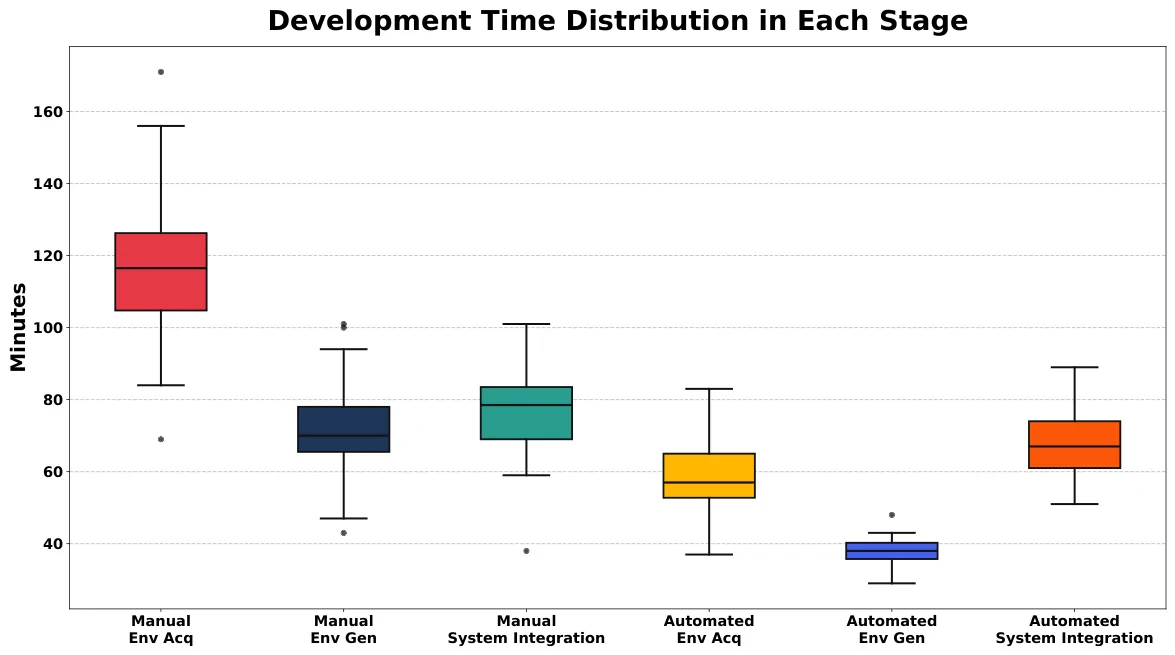
Participant-level distributions of development time for the manual and automated workflows across the three development stages.

Figure 8 presents the participant-level distributions of development time across workflows and stages. The automated workflow showed lower central values at all three stages and lower descriptive variability, particularly during environment acquisition and environment generation.

### 5.3. NASA-TLX Perceived Workload Results

Participants completed the NASA Task Load Index (NASA-TLX) questionnaire after each workflow condition. The unweighted Raw TLX score was calculated for each participant and condition as the arithmetic mean of the six subscale ratings: mental demand, physical demand, temporal demand, performance, effort, and frustration.

**Figure 9:**
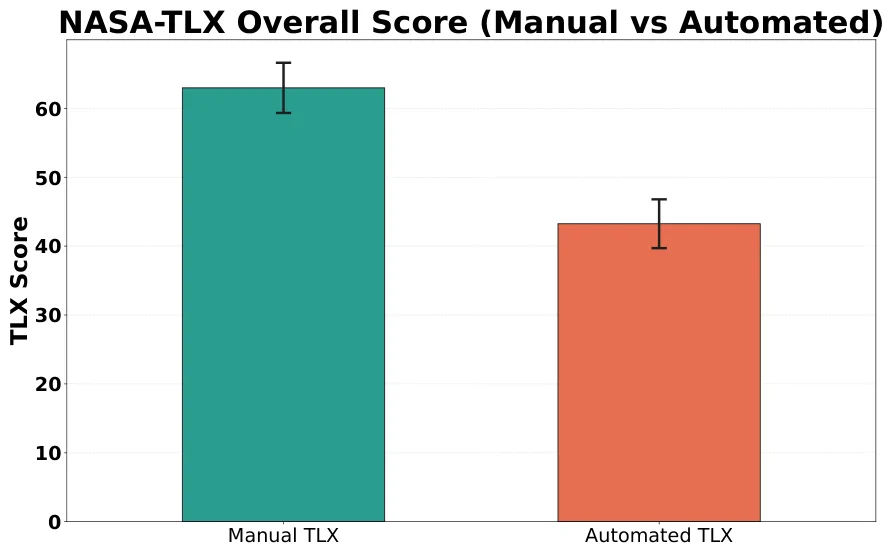
Comparison of participant-level overall Raw TLX scores between the manual and automated workflows.

The participant-level Raw TLX scores indicated lower overall perceived workload under the automated workflow than under the manual workflow, as illustrated in Figure 9. The manual workflow produced a mean Raw TLX score of 62.97 (SD = 3.64), whereas the automated workflow produced a mean score of 43.25 (SD = 3.54).

A paired-sample *t*-test showed a significant difference in overall perceived workload between the two workflows, t(47)=24.52, p<0.001. The mean manual-minus-automated difference was 19.72 points (95% CI [18.10, 21.34]), corresponding to an approximately 31.3% reduction relative to the manual-workflow mean. The standardized paired effect size was $d_{z}=3.54$.

To describe the workload dimensions contributing to the overall pattern, the six NASA-TLX subscale scores were summarized separately. Table 4 presents the mean and standard deviation of each subscale under both workflow conditions.

Descriptively, the manual workflow produced higher mean scores for mental demand, temporal demand, perceived performance, effort, and frustration, whereas the automated workflow produced a higher mean physical-demand score. For the performance subscale, a higher score represents poorer perceived performance. Because separate inferential tests were not conducted for the individual subscales, these differences are interpreted descriptively and are not presented as independently established effects.

### 5.4. Spatial Alignment Validation Results

A separate spatial alignment validation was conducted by the research team using one manual implementation and one automated implementation created specifically for this evaluation. The validation used 13 predefined anchor points distributed across navigation-relevant structural locations, including door centers, corridor intersections, wall corners, and navigation turning points. Each anchor was evaluated through five repeated localization or alignment attempts for each implementation, producing 65 measurements per implementation and 130 measurements in total.

Table 5 presents the mean and standard deviation of the three-dimensional Euclidean alignment error calculated from the five repeated attempts at each anchor. Across all 65 measurements per implementation, the manual implementation produced an overall mean positional error of 19.14 cm (SD = 1.07 cm), whereas the automated implementation produced an overall mean error of 22.47 cm (SD = 1.21 cm).

For inferential analysis, the five repeated measurements were averaged within each anchor and implementation. This aggregation produced 13 paired anchor-level mean errors, with the anchor locations serving as the units of inferential comparison. The Shapiro–Wilk test did not indicate a departure from normality for the paired difference scores (p=0.597). A paired-sample *t*-test showed that the automated implementation had a significantly higher mean anchor-level alignment error than the manual implementation, t(12)=28.39, p<0.001. The mean automated-minus-manual difference was 3.33 cm (95% CI [3.07, 3.58] cm).

These results demonstrate a consistent difference between the two researcher-generated implementations at the sampled anchor locations. The result characterizes the evaluated implementations within the tested environment and should not be interpreted as a participant-level comparison of spatial accuracy or as evidence that the same difference would occur across all implementations produced using either workflow.

Both evaluated implementations supported the predefined basic navigation functions in the tested environment. This observation was not treated as a measured navigation-success rate or as a general acceptance threshold for spatial accuracy in other AR navigation systems or deployment environments.

### 5.5. Exploratory Analysis of Participant Experience

A post hoc exploratory analysis examined whether participant-level automated-workflow benefits differed according to prior AR/Unity or 3D modeling experience. Benefit scores were calculated as the manual-workflow value minus the corresponding automated-workflow value for total development time and overall Raw TLX. Positive scores therefore represented time savings or workload reductions under the automated workflow. The resulting benefit scores were compared between experience subgroups using two-tailed Welch independent-samples *t*-tests. Holm correction was applied across the four exploratory comparisons.

The mean development-time benefit was 100.64 min (SD = 31.23) for participants with two years of AR/Unity experience (n=11) and 101.32 min (SD = 28.10) for those with one year of experience (n=37). The difference was not statistically reliable, t(15.15)=−0.07, unadjusted p=0.949, Holm-adjusted p=1.000. Participants with prior 3D modeling experience (n=35) had a mean benefit of 102.57 min (SD = 29.39), compared with 97.38 min (SD = 26.69) among those without prior experience (n=13). This difference was also not statistically reliable, t(23.56)=0.58, unadjusted p=0.566, Holm-adjusted p=1.000.

For overall Raw TLX, participants with two years of AR/Unity experience had a mean benefit of 23.88 points (SD = 6.63), whereas those with one year of experience had a mean benefit of 18.49 points (SD = 4.64). The 5.39-point difference was nominally significant before correction, t(13.05)=2.52, unadjusted p=0.025, but was not statistically reliable after Holm correction (p=0.102). Participants with and without prior 3D modeling experience had mean Raw TLX benefits of 19.62 points (SD = 5.02) and 19.99 points (SD = 7.09), respectively. This difference was not statistically reliable, t(16.68)=−0.17, unadjusted p=0.867, Holm-adjusted p=1.000.

The exploratory analysis did not provide statistically reliable evidence, after correction for multiple comparisons, that development-time or overall Raw TLX benefits differed according to the measured experience variables. This absence of statistically reliable subgroup differences should not be interpreted as evidence of equivalence across expertise levels because the subgroup sizes were unequal, the range of experience was restricted, and the sample did not include a distinct professional-developer group.

## 6. Discussion

The participant experiment showed that automated cloud-based mapping reduced both development time and overall perceived workload relative to manual 3D modeling under the tested conditions. The automated workflow reduced mean total development time by approximately 38.3%. Significant reductions occurred at all three development stages, although the differences were substantially larger during environment acquisition and generation than during system integration. This pattern reflects the shift from physical measurement and explicit geometric modeling toward image acquisition and cloud-based map generation. It is also consistent with prior work positioning feature-based mapping and visual localization as alternatives to fully manual environment reconstruction [7,14,17].

The automated workflow also reduced the mean overall Raw TLX score by approximately 31.3%. Descriptively, participants reported lower mental demand, temporal demand, perceived performance difficulty, effort, and frustration under the automated workflow, while physical demand was higher. The higher physical-demand score is consistent with the need to move through the environment while capturing images from multiple viewpoints. Because the individual subscales were not tested inferentially, these dimensional differences should be interpreted descriptively; the inferential workload conclusion applies only to the participant-level overall Raw TLX comparison.

The separate researcher-conducted spatial validation identified a trade-off in the two implementations generated specifically for that evaluation. Across 13 predefined anchor locations, the automated implementation produced a mean three-dimensional positional error of 22.47 cm, compared with 19.14 cm for the manual implementation, corresponding to a significant mean anchor-level difference of 3.33 cm. This result characterizes one implementation per workflow in the tested environment and should not be interpreted as a participant-level comparison or as evidence that the same difference would occur across all implementations. Both implementations supported the predefined basic navigation functions, but this observation does not establish a general spatial-accuracy threshold or a measured navigation-success rate.

The spatial validation was limited to point-based alignment measurements at 13 locations in a single indoor environment. Trajectory-level pose error, localization stability, tracking failures, relocalization success, mapping completeness, mapping reliability, and navigation success were not independently measured. The experiment also did not collect standardized incident logs or visual records of successful and failed mapping or navigation cases. Consequently, the available data support an anchor-level comparison of the evaluated implementations but not a comprehensive localization-performance or qualitative failure analysis.

The experiment was conducted on one 1084 $m^{2}$ campus-building floor using one smartphone model and one automated mapping platform. Environmental characteristics such as visual texture, repetitive structures, illumination, dynamic activity, occlusion, and image coverage were not independently manipulated, although these factors can affect image-based localization [45,46]. Passive network-transmission and server-processing waits were excluded from development time, so the reported time differences represent active workflow effort rather than complete end-to-end elapsed time. The evaluated cloud-generation workflow depended on network availability during map creation, but this should not be interpreted as requiring continuous connectivity during subsequent localization [33]. Computational-resource consumption, service cost, update effort, and scaling behavior were also not measured and therefore remain practical considerations rather than demonstrated comparative outcomes.

Finally, the relatively homogeneous participant population limits generalization of the development-time and workload findings. The exploratory subgroup analysis did not provide statistically reliable evidence, after Holm correction, that workflow benefits differed according to the measured AR/Unity or 3D modeling experience variables. This absence of significant subgroup differences does not demonstrate equivalence across expertise levels because the subgroups were unequal, the experience range was restricted, and professional developers were not separately represented. Future studies should evaluate multiple indoor environments, more diverse developer populations, different devices and mapping platforms, systematic environmental variations, trajectory- and task-level localization outcomes, and alternative approaches such as LiDAR-assisted or hybrid reconstruction workflows.

## 7. Conclusions

This study presented a controlled, counterbalanced within-subject comparison of manual 3D modeling and automated cloud-based image mapping for AR indoor navigation environment preparation. Under the tested conditions, the automated workflow reduced the mean total development time by approximately 38.3% and the mean overall Raw TLX by approximately 31.3%. Development-time reductions were significant at all three stages but were most pronounced during environment acquisition and generation. Although overall perceived workload was lower, the automated workflow produced a descriptively higher physical-demand score associated with the environment-capture process. A separate researcher-conducted spatial validation revealed an implementation-level trade-off. Across repeated localization and alignment attempts at 13 predefined anchor locations, the automated implementation produced a mean three-dimensional positional error of 22.47 cm, compared with 19.14 cm for the manual implementation. The significant mean difference of 3.33 cm indicates that the observed efficiency and workload benefits were not accompanied by equivalent spatial alignment in the two evaluated implementations. These findings are limited to one indoor environment, one automated mapping platform, one smartphone model, a relatively homogeneous participant population, and one researcher-generated implementation per workflow for spatial validation. They should therefore be interpreted as workflow trade-offs observed under the tested configuration rather than generalized performance differences across all AR navigation environments, developer populations, or spatial-mapping technologies. Future studies should include more diverse environments and participants, multiple independently generated implementations, additional localization and navigation metrics, and comparisons with LiDAR-assisted and hybrid reconstruction approaches.

## Figures and Tables

**Table 1:** Comparison of representative spatial mapping and localization approaches relevant to AR environment preparation.

Approach	Primary Input	Spatial Representation	Primary Role	Key Consideration
Manual 3D modeling	Measurements/floor plans	Explicit 3D geometry	Environment modeling and navigation configuration	High manual modeling effort
Apple RoomPlan	Camera + LiDAR	Structured parametric geometry	Indoor room reconstruction	Requires compatible LiDAR-equipped Apple devices
ARCore Cloud Anchors	Camera-based visual features	Persistent feature-based anchors	Persistent/shared AR content	Cloud connectivity and anchor-oriented use
Azure Spatial Anchors	Camera-based spatial observations	Persistent spatial anchors	Cross-device persistent AR content	Service retired in November 2024
Immersal [33]	Camera images	Feature-oriented visual map	Visual relocalization and spatial registration	Cloud-based map generation; no dedicated depth sensor required

**Table 2:** Total development time for the manual and automated environment-preparation workflows.

Workflow	Mean (min)	SD (min)
Manual 3D modeling	264.5	27.3
Automated mapping	163.3	14.9

**Table 3:** Development time by workflow and development stage.

Stage	Manual Mean (min)	SD (min)	Automated Mean (min)	SD (min)
Environment acquisition	116.4	19.0	57.7	9.9
Environment generation	71.1	13.2	38.0	3.8
System integration	77.0	11.3	67.6	9.3

**Table 4:** Descriptive NASA-TLX subscale scores for the manual and automated workflows.

Dimension	Manual Mean	SD	Automated Mean	SD
Mental demand	82.71	11.44	46.27	8.46
Physical demand	35.23	8.69	64.69	11.69
Temporal demand	72.42	7.89	44.08	6.78
Performance	51.42	11.09	25.92	9.05
Effort	79.69	9.80	43.54	9.15
Frustration	56.38	9.16	35.00	9.14

**Table 5:** Three-dimensional spatial alignment error across repeated attempts at the 13 anchor locations.

Anchor	Manual Mean ± SD (cm)	Automated Mean ± SD (cm)	Automated–Manual Difference (cm)
1	18.14 ± 1.00	21.68 ± 0.97	3.55
2	18.94 ± 0.92	22.42 ± 0.49	3.48
3	19.73 ± 0.65	22.96 ± 1.59	3.23
4	19.11 ± 0.76	21.90 ± 0.89	2.78
5	19.12 ± 1.66	22.96 ± 1.72	3.84
6	18.30 ± 1.06	22.35 ± 1.30	4.05
7	19.14 ± 1.05	22.79 ± 1.42	3.65
8	19.61 ± 0.88	22.87 ± 1.48	3.26
9	19.33 ± 1.43	22.88 ± 1.43	3.55
10	19.28 ± 0.82	22.14 ± 0.50	2.86
11	18.97 ± 1.46	22.41 ± 1.53	3.44
12	19.42 ± 0.87	22.12 ± 0.77	2.70
13	19.69 ± 0.73	22.57 ± 1.43	2.88
Average	19.14 ± 1.07	22.47 ± 1.21	3.33

## Data Availability

The original contributions presented in this study are included in the article. Further inquiries can be directed to the corresponding authors.
